# Efficacy of native cyclopoid copepods in biological vector control with regard to their predatory behavior against the Asian tiger mosquito, *Aedes albopictus*

**DOI:** 10.1186/s13071-022-05460-y

**Published:** 2022-10-01

**Authors:** Isabel Pauly, Oliver Jakoby, Norbert Becker

**Affiliations:** 1 Faculty of Bioscience, University of Heidelberg, Im Neuenheimer Feld 230, 69120 Heidelberg, Germany; 2 Effect Modelling and Statistics, RIFCON GmbH, Goldbeckstraße 13, 69493 Hirschberg, Germany; 3 Institute of Dipterology (IfD), Gesellschaft Zur Förderung Der Stechmückenbekämpfung E.V., Georg-Peter-Süß-Str. 3, 67346 Speyer, Germany

**Keywords:** *Aedes albopictus*, Copepoda, Vector control, Arboviruses, *Megacyclops viridis*, Predatory potential

## Abstract

**Background:**

The control of the Asian tiger mosquito *Aedes albopictus* (Diptera: Culicidae) is crucial owing to its high vector competence for more than 20 arboviruses—the most important being dengue, chikungunya and Zika virus. *Aedes albopictus* has an enormous adaptive potential, and its invasive spreading across urban and suburban environments poses challenges for its control. Therefore, all suitable, cost-effective and eco-friendly control tools should be put into practice. In this context, cyclopoid copepods are already known as effective predators of mosquito larvae. This study reports an essential preliminary step towards the integration of copepods into the vector control strategy in Germany, in order to provide a sustainable tool in an integrated control strategy based on the elimination or sanitation of breeding sites, the use of formulations based on *Bacillus thuringiensis israelensis* (*Bti.*) and the sterile insect technique (SIT).

**Methods:**

The predatory potential of native cyclopoid copepods, namely the field-derived species *Megacyclops viridis* (Crustacea: Cyclopidae), was examined against the larvae of *Ae. albopictus*, and for comparison, against the larvae of the common house mosquito, *Culex pipiens* sensu lato (Diptera: Culicidae). The use of different larval instars as prey, and various predator-to-prey ratios, were examined under laboratory and semi-field conditions. The compatibility of *Bti.* applications along with the use of copepods was assessed in the laboratory.

**Results:**

High predation efficiency of *M. viridis* upon first-instar larvae of *Ae. albopictus* was observed under laboratory (up to 96%) and semi-field conditions (65.7%). The copepods did not prey upon stages further developed than the first instars, and in comparison with *Ae. albopictus*, the predation rates on the larvae of *Cx. pipiens* s.l. were significantly lower.

**Conclusions:**

The results indicate a high predation potential of *M. viridis* against *Ae. albopictus* larvae, even though strong larval stage and mosquito species preferences were implicated. The integration of copepods as a promising biocontrol agent to the vector control strategy in Germany is therefore highly recommended, especially because of the excellent compatibility of copepods with the use of *Bti*. However, further research is required, concerning all the probable parameters that may impact the copepod performance under natural conditions.

**Graphical Abstract:**

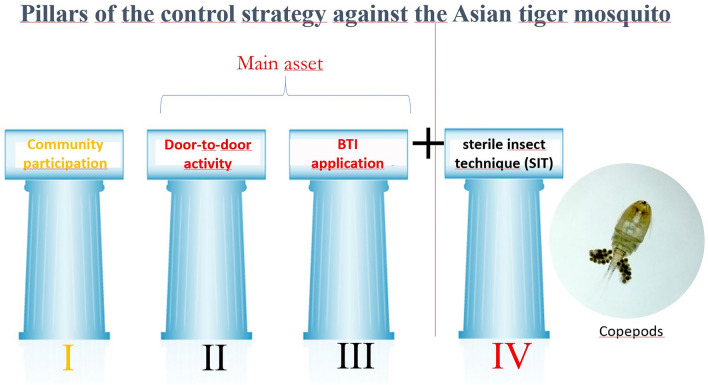

**Supplementary Information:**

The online version contains supplementary material available at 10.1186/s13071-022-05460-y.

## Background

In the past, mosquito-borne diseases were almost exclusively associated with the tropical and subtropical regions of the Southern Hemisphere. However, the influence of global warming and globalisation have strongly accelerated the risk of the introduction and establishment of invasive mosquito species, and their corresponding pathogens, in different parts of the world [1]. Of the group of invasive species that have successfully expanded their habitat beyond their former dispersal areas, the Asian tiger mosquito, *Aedes albopictus* (Skuse 1895)—originally inhabiting the Asia-Pacific region [2]—merits careful attention owing to its high distributional potential and vector competence, especially for arboviruses like dengue, chikungunya and Zika virus [3]. Since 2007, adult *Ae. albopictus* mosquitoes that have travelled from the Mediterranean area, where their populations are abundant, have been continuously trapped at resting stations along highways in southern Germany [4, 5]. In 2014, the first established and reproducing population of the Asian tiger mosquito was reported [6]. From then on, the number of identified populations has increased to more than 20 locations in Southwest Germany, as well as some more northern regions of Germany. The species is now considered as firmly established in the country [7, 8].

Several circumstances have led to the ability of *Ae. albopictus* to thrive globally. Firstly, it possesses a great capacity to adapt to temperate geographical regions, and its distribution is additionally favoured by the rising temperatures caused by global warming [9]. Secondly, the adult females of this species are able to produce desiccation-resistant eggs, which can survive long periods of transport, absence of water, and low temperatures [1]. In particular, the embryonic diapause of their winter eggs from October to April has allowed *Ae. albopictus* mosquitoes to avoid elimination during Central European winters, and enabled them to establish populations even under moderate climate conditions [10].

The ease of its introduction, coupled with its ecological variability, supports the settling of this mosquito and makes it highly unlikely that the mosquito will ever be entirely eradicated from Germany. Even though the climatic conditions have been unfavourable for arbovirus transmissions up to the present time, rising temperatures and the increasing numbers of mosquitoes underline the importance of combating these invasive vectors. Therefore, the integrated vector control program in Southwest Germany aims to reduce the risk of potential future arbovirus transmissions by suppressing the vector populations [1].

Modern vector control strategies against *Ae. albopictus* in Germany focus on reducing the vector populations without using synthetic or chemical insecticides, in order to avoid a negative impact on the biodiversity of non-target organisms. At present, the control strategy comprises three pillars: (a) community participation (CP) based on the elimination or sanitation of breeding sites with the use of fizzy *Bacillus thuringiensis israelensis* (*Bti*) tablets (Culinex^®^ Tab plus; (b) door-to-door (DtD) control by trained staff applying high doses of a *Bti* water-dispersible granular formulation (Vectobac^®^ WG) aiming for a long-lasting killing effect; and (c) the sterile insect technique (SIT) to eliminate remaining *Ae. albopictus* populations [11]. The application of formulations based on *Bti* guarantees nematoceran-specific control and therefore only kills mosquitoes in the breeding sites of *Ae. albopictus*, leaving non-target organisms unharmed [12–14]. Another important feature of *Bti* is that no resistance has developed so far, even after more than 40 years of its wide-scale application against mosquitoes [15]. However, *Bti* formulations offer a sufficient killing rate for only a limited number of weeks, even at higher-dose applications [14], and therefore a continuous and repeated application of the larvicide is required during the mosquito breeding season (April to November) [16]. This disadvantage can potentially be compensated by the simultaneous inoculation of natural predators to the breeding sites, to feed upon newly hatched larvae as the impact of *Bti* wanes. These predators should therefore maintain stable populations within bodies of water, creating a sustainable, long-term vector control [13].

The predatory behaviour of copepod crustaceans (Crustacea: Copepoda) against larvae of *Aedes* mosquitoes was primarily observed and described by Riviere and Thirel in 1981 [17]. Nowadays, these copepods are considered the most efficient invertebrate predators of mosquito larvae and are a promising tool in the control of container-breeding mosquitoes [18–20]. Out of the three copepod orders that inhabit fresh waters, only the Cyclopoida demonstrate a carnivorous diet [21]. Hence, in the context of this study, the term “copepods” only refers to cyclopoid species. In particular, cyclopoid genera with a sufficiently large body size prefer to feed upon small aquatic animals, including mosquito larvae [22, 23]. However, they mostly show an omnivorous diet, allowing them to establish stable populations in nearly every aquatic habitat [21, 24]. In addition, copepods combine a striking number of advantages compared with other potential larval predators [1]. Their ecological variability, robustness, tolerance to a wide temperature range and small body size (1–3 mm) permit their survival even in small water containers, which are known to be common breeding sites for *Ae. albopictus* and *Culex pipiens* sensu lato [21]. Assuming a favourable nutrient supply and permanent water availability, they can maintain stable populations over several years in artificial containers [19]. Because of these favourable qualities of copepods, the predatory efficiency of various cyclopoid species against the larvae of different types of mosquito—mainly focusing on the genera *Aedes* and *Culex*—has been intensively examined over the last few decades under laboratory, semi-field and field conditions [20, 21, 25]. Indigenous cyclopoid species of different geographical regions were capable of significantly reducing larval prey up to 99–100% in laboratory assays in Asia, the United States, various countries of South America, and Italy [19, 22, 26–29].

The largest cyclopoid genera (> 1.4 mm body length), such as *Megacyclops* or *Macrocyclops* (Cyclopoida: Cyclopidae), were identified as the most effective predators, indicating a positive correlation between their body size and predatory efficiency [30]. Therefore, copepods mainly prey on first-instar larvae, and to a lesser extent on second-instar larvae, as the more developed larval stages exceed the maximum size of potential copepod prey [31]. Furthermore, they show significant differences in their preferences towards different mosquito species [21]. In general, copepod species proved to prey more efficiently upon *Aedes* than *Culex* larvae, indicating their varying prey preferences [20].

When considering copepods as biocontrol agents, only indigenous copepod species should be considered, since they do not pose any threat to the local ecosystem and fauna [32, 33]. In addition, it was even noted that different populations of the same copepod species exhibit biological variability regarding their predatory efficiency [34]. To include the varying predatory efficiency of copepod species and populations, it is therefore essential to first examine the utility of indigenous copepod species against the mosquito in question, in this case *Ae. albopictus*, under laboratory and field conditions. The promising studies conducted in the United States, Asia and South America are yet to be sufficiently evaluated in European countries [20]. A few studies have addressed the use of copepods against *Aedes* species in the UK [30, 35] and Italy [32], while German native copepod species have been evaluated for their efficacy against the invasive mosquito *Aedes japonicus* (Theobald 1901) by Früh et al. [36], but to our knowledge, they have not yet been tested against *Ae. albopictus* or *Cx. pipiens* s.l.

Therefore, in this study we assessed the effectiveness of locally abundant copepods as biological control agents of *Ae. albopictus* and *Cx. pipiens* s.l. larvae, in laboratory and semi-field tests, which included the species identification of the copepods. This represents an essential preliminary step towards incorporating indigenous copepods into the local vector control strategy in practice, and creating a sustainable, cost-effective and eco-friendly mosquito control. According to numerous studies that support the potential of copepods, it was hypothesised that local, field-derived copepods would prey upon first-instar larvae of both mosquito species, but to a lesser extent upon *Cx. pipiens* s.l., as well as further-developed larval stages.

## Methods

### Study material

#### Field-derived copepods

All copepods were collected in the field from May to July 2021 from a pond in Philippsburg, Germany (49°14′44.6″N 8°26′45.0″E). The copepods were obtained from the shallow region of the water sources, including the aquatic vegetation area, using a long-handled fine plankton net (mesh size 100 µm). The collected water organisms were transferred to glass jars and transported in Styrofoam boxes to the laboratory for species determination and sorting of the relevant species.

Sorting took place under laboratory conditions no later than 1 day after the collection. The water samples were transferred to sorting trays (approximately 500 ml), and the cyclopoid copepods with the largest relative body size, especially those carrying egg sacs, were selected and pipetted into plastic boxes filled with approximately 600 ml of fresh tap water (pH 7.4, 20 °C, 521 µS; valid for all subsequent experiments). The selection of the copepods based on their body size ensured the use of females of commonly bigger cyclopoid genera instead of smaller males or copepodids [36]. Field-derived copepods were stored until use at room temperature (24 ± 1 °C) and protected from direct insolation. The maximum capacity per box was kept below 150 individuals to ensure longevity. The copepods were fed one spatula-tip of fish food powder (TetraMin Baby^®^, Tetra GmbH) per box, every 3–5 days. In addition, residues were removed and fresh water was added, when needed. Copepods were stored for no longer than 7 weeks.

#### Mosquito larvae

Eggs of *Ae. albopictus* were obtained from colonies run by the Institute of Dipterology IfD/*Gesellschaft zur Förderung der Stechmückenbekämpfung* (GFS; Speyer, Germany) and the *Centro Agricoltura Ambiente “G. Nicoli”* (CAA; Crevalcore, Italy). Approximately 24 h before the experiments, the eggs were flooded with fresh tap water at room temperature (24 ± 1 °C) to allow the emergence of the larvae. Egg rafts of *Cx. pipiens* s.l. were collected from rain barrels in the garden of IfD approximately 24 h before the experiment (Speyer, Germany). The egg rafts were kept at room temperature (24 ± 1 °C) in water derived from the breeding sources until hatching of the larvae.

Larvae that were not used as freshly hatched larvae were reared at room temperature (24 ± 1 °C) in glass jars covered with nets. The larvae were fed one spatula-tip of fish food every second day, until the necessary developmental stage for further experiments was reached.

### Experimental setting

Field-derived gravid or non-gravid female copepods of similar size and appearance were used in the experiments. After the experiments, the copepods were killed in alcohol and preserved in 70% ethanol, and treated with one drop of glycerol for precise species identification [36], according to the fixation and preparation process described by Einsle [24].

### Laboratory experiments

#### Assessment of the predation efficiency of the copepods against different larval instars of *Ae. albopictus*

The predation rates of copepods were separately assessed for the first three larval developmental stages. The experiments on first-instar larvae were performed three times with five replicates for each control and treatment groups (number of replicates per group: *n* = 15). The predation efficiency upon second and third developmental stages were only tested once (*n* = 5).

Ten transparent plastic boxes (1.1 L), each filled with 500 ml of fresh tap water, were used as experimental containers. Five larvae of the same mosquito species at the same corresponding development stage were transferred to each container. Based on the procedures reported by Chansang et al. [37] and Rey et al. [31], one adult female copepod per box was introduced to five of the containers to establish a 1:5 ratio between prey and predator. Boxes containing only larvae served as a control to account for background prey mortality. One spatula-tip of powdered fish food was added to each box to provide nutrition. All laboratory experiments were carried out at room temperature (24 ± 1 °C). Remaining living larvae were counted after 24 h and 48 h, to determine the number of killed larvae. Larval remnants (head capsules, fragments or mangled bodies) were removed, identified and counted under a binocular microscope (Motic SMZ 171-BP^®^) as proof of the predatory behaviour [29, 31, 36].

The predation rate was defined as the number of killed larvae per copepod per day [38], and the predation efficiency was calculated according to Abbott’s formula [30, 39]:

$${\text{Predation\,efficiency }} = \frac{Number\,alive\,in\,control - Number\,alive\,in\,treatment }{{Number\,alive\,in\,control}} \left( {{1}00} \right)$$

Following the same procedure as described above, ad libitum feedings were performed on first-instar larvae of *Ae. albopictus* (*n* = 15), only differing in the amount of prey that was offered to the copepods. Instead of five larvae, 24 first-instar larvae were exposed to one adult female copepod. This experiment was not conducted for the further developed larval stages.

#### Simultaneous predation efficiency analysis against *Ae. albopictus* and *Cx. pipiens* s.l. larvae

One day prior to the experiment, five transparent buckets (10 L), each filled with 8 L of fresh tap water, were each inoculated with four adult female copepods for acclimatisation and starvation. Twenty-four hours later, 10 first-instar larvae of *Ae. albopictus* and 10 first-instar larvae of *Cx. pipiens* s.l. were added to each bucket, so that a 1:5 ratio was established. Five buckets with the same number of larvae, but without copepods, served as control. Larvae were fed two spatula-tips of fish food per bucket.

The evaluation was carried out 5 days after the introduction of the larvae, when the larvae had reached a developmental stage of late second- to third-instar larvae. To differentiate the species of the remaining larvae, they were collected from the buckets and identified under the binocular microscope. The main characteristic for differentiation was the siphon, which differs in shape and length between the two species [1].

#### Assessment of the compatibility of the use of *Bti* and copepods in an integrated control strategy

Three different concentrations (1, 10, 50 ppm) of *Bti* (3000 ITU/mg, VectoBac WG^®^, Lot No. 320917PG30) were prepared by diluting the corresponding amount of 50 ppm *Bti* stock solution (0.15 g *Bti* powder, 3.00 L H_2_O) with an appropriate amount of fresh tap water, to reach a final experimental volume of 200 ml. One part per million was chosen as the lowest concentration, since it represents a concentration of 1000 µg *Bti*/L, which is commonly applied in mosquito control programs [14]. The effect of *Bti* was tested on either five *Cx. pipiens* s.l. first-instar larvae (as positive control) or five adult female copepods. The negative controls contained 200 ml of tap water and five adult female copepods.

The assays for the three groups (positive control, negative control and copepods in *Bti* water) at the three different concentrations were performed in triplicate, which produced a total of 27 assays (*n* = 3). They were performed in transparent plastic beakers, to which a spatula-tip of powdered fish food was added, respectively. The results were recorded 24 h and 48 h after the introduction of the organisms.

### Assessment of the predation rate of *Ae. albopictus* larvae in semi-field tests

Ten rain barrels (70 L), approximately three-quarters full with tap water, were installed at room temperature (24 ± 1 °C) 1 week prior to the experiment in a semi-outdoor environment that did not bear any risk of the mosquitoes escaping (laboratory premises of GFS, Speyer, Germany).

One day before the experiment, 10 adult female copepods were added to five of the 10 rain barrels, respectively, to account for acclimatisation and starvation. Twenty-four hours later, 50 newly hatched larvae of *Ae. albopictus* were inserted to each rain barrel, resulting in a 1:5 predator-to-prey ratio. The other five rain barrels served as a control, containing only larvae. Ten spatula-tips of fish food were added to each barrel. Feeding was performed every second day over the whole duration of the experiment, gradually increasing the amount of available food by an additional five spatula-tips at every feeding.

Five days after the installation, the number of surviving prey was counted by extracting the larvae from the barrels into water-filled sorting trays and pipetting them one by one into another water-filled container. In the case of the controls, all counted larvae were killed to avoid further development. In contrast, only the third- and fourth-instar larvae from the barrels containing copepods were disposed of, since it was unlikely that they would have been eaten afterwards, and further development was to be avoided as in the controls. All first- or second-instar larvae were poured back into the respective barrels with copepods to account for the possibility that the copepods would still kill them in the remaining experimental time. Following this procedure, counting was performed for 2 more days. In addition, all copepods, copepodids and larval fragments that were unintentionally collected with the net were enumerated.

### Species identification of field-derived copepods

For species identification, 12 preserved copepods from the laboratory predation efficiency experiments were randomly selected, dissected and identified under the microscope (Motic Panthera C2^®^ microscope; *n* = 12) according to the keys of Błędzki and Rybak [40] and Einsle [24]. For final species identification, the fourth and fifth paired swimming legs (P4 and P5), the furca, as well as the specific ecology, incidence and body size of the different species, were considered. Each of those characteristic body features were photographed with a Canon EOS 90D^®^ camera.

### Statistical analysis

The predation rates of copepods in the different experiments were analysed using generalised linear (mixed) models [GL(M)Ms]. The response variable, i.e. the survival of larvae, is binomially distributed (alive or dead). Hence, generalised linear (mixed) models with a binomial family using a logit link are applied.

As fixed factors in the GL(M)Ms, the different treatment groups (i.e. with or without predation, low and high density or predation in different prey species) were used as explanatory variable. For logistic reasons, in all experiments (except of the semi-field study) a set of five replicates per treatment needed to be repeated three times in a row to obtain the overall 15 replicates. Hence, variable the “batch” (i.e. number of experiment repetition) was used in all GL(M)Ms as a random factor (model 1). Moreover, for the analysis of the simultaneous predation both prey species were kept in the same bucket. Therefore, in this analysis, the bucket number was also included as a random factor nested in batch (model 2). The semi-field experiment was analysed using a generalised linear model (GLM), as it was conducted only once with five replicates per treatment (model 3). The model assumptions were evaluated using diagnostic plots.

All statistical analyses were conducted with the statistical and programming environment R (version 4.2.0) [41]. The GL(M)Ms were fitted using functions of the *lme4* package [42].

Differences between tested groups were considered significant if the *P*-value was  ≤ 0.05, indicated in the figures by asterisks (*P < 0.05; **P < 0.01 or ***P < 0.001). In the results section data values were expressed as means ± standard deviation (SD).

## Results

### Species-identification of field-derived copepods

Eleven of the 12 randomly selected field-derived copepods from the laboratory trials were identified as *Megacyclops viridis* (Jurine 1820; 2.5 ± 0.14 mm). One copepod could be allocated to the genus *Megacyclops* without further specification of the species, due to the lack of its furca after the dissection.

### Predation efficiency analysis against *Ae. albopictus* first-instar larvae

**Fig. 1:**
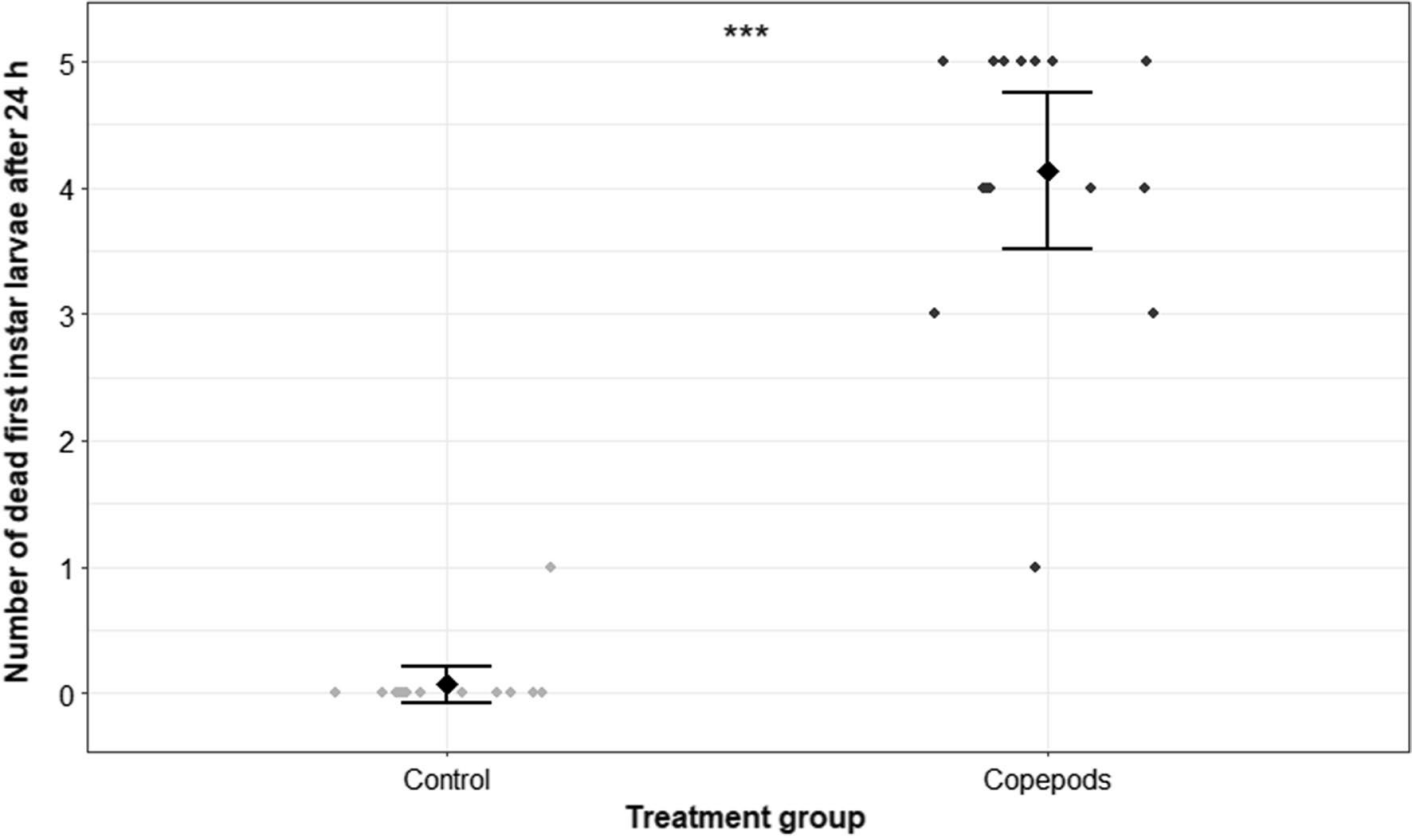
Number of dead *Ae. albopictus* first-instar larvae after exposing five larvae per replicate (*n* = 15) to either no (control) or one adult copepod for 24 h. Each point refers to one experimental replicate. The rhombus indicates the mean of dead larvae in each group. Statistically significant difference between groups, according to a GLMM analysis (***P < 0.001), is indicated by asterisks. Error bars represent the 95% confidence interval

Compared with the control experiments, *M. viridis* significantly reduced the number of living prey during a 24 h predation period, when five first-instar larvae of *Ae. albopictus* were offered to one adult female copepod (GLMM model 1; *P* < 0.001; *n* = 15). In the trials without copepods, only one larva died during the observation period, whereas an average of 4.8 ± 0.4 larvae killed by the copepods was the highest value observed after 24 h in one experimental run (*n* = 5) of the treatment groups, which amounts to a predation efficiency (percentage of larvae consumed according to Abbott’s formula) of 96% (Table 1). On average, 4.1 ± 1.1 larvae were dead after 24 h in the copepod treatment group, while only 0.1 ± 0.3 were found dead in the control group (Fig. 1). The copepods reduced the larval survival to an even greater extent after a predation period of 48 h compared with the control groups (GLMM model 1; *P* < 0.001; *n* = 15). At this time, 4.2 ± 0.7–5.0 ± 0.0 larvae were killed by the copepods in the three experimental runs (*n* = 5 each), which consequently leads to predation efficiency ranging from 84.0% to 100.0% (Table 1).

**Table 1:** Number of dead *Ae. albopictus* first-instar larvae after exposing five larvae per replicate (*n* = 15) to one adult copepod. Predation efficiency was calculated according to Abbott’s formula

	After 24 h			After 48 h		
Run No° (*n* = 5)	Mean ± SD (predation rate)	Range	Predation efficiency [%]	Mean ± SD (predation rate)	Range	Predation efficiency [%]
1	4.2 ± 0.7	3–5	84.0	4.8 ± 0.4	4–5	96.0
2	4.8 ± 0.4	4–5	96.0	5.0 ± 0.0	5	100.0
3	3.4 ± 1.4	1–5	68.0	4.2 ± 0.7	3–5	84.0
Overall (*n* = 15)	4.13 ± 1.13		82.7	4.67 ± 0.62		93.3

*n* number of replicates, *No°* sample number, *SD* standard deviation

According to the ad libitum predation experiments, in which 24 larvae were offered to one adult female copepod, between nine and 21 *Ae. albopictus* first-instar larvae were found dead within 24 h (Table 2). This translates to a predation efficiency of up to 63.5% in 24 h in one experimental run (*n* = 5) (Table 2). The predation rate, defined as the average number of killed larvae per copepod per day, ranged from 12 ± 2.7 to 15.6 ± 5.2 (Table 2). The predation efficiency and predation rate increased with a longer predation period of 48 h. In three of the 15 replicates, the copepod killed 22–24 larvae within 48 h. Considering all three experimental runs (*n* = 5 each), the predation efficiency amounted to 87% after 48 h in the highest run (Table 2). Overall, the prey survival in the controls (all 15 replicates) amounted to 93.6% ± 6.07%, so it was obvious that the prey mortality in the treatment groups was caused by predation by the copepods (GLMM model 1; *P* < 0.001; *n* = 15). Larval fragments were detected in the experimental containers as additional proof of copepod feeding. The hunting and consumption process by the copepods was repeatedly observed in real time, and was also recorded on video.

**Table 2:** Number of dead *Ae. albopictus* first-instar larvae after exposing 24 larvae per replicate (*n* = 15) to one adult copepod. Predation efficiency was calculated according to Abbott’s formula

	After 24 h			After 48 h		
Run No° (*n* = 5)	Mean ± SD (predation rate)	Range	Predation efficiency [%]	Mean ± SD (predation rate)	Range	Predation efficiency [%]
1	15.2 ± 2.9	13–20	63.0	20.0 ± 2.7	17–24	83.2
2	12 ± 2.7	9–16	47.8	19.6 ± 3.2	14–22	80.0
3	15.6 ± 5.2	10–21	63.5	21.2 ± 4.1	15–24	87.0
Overall (*n* = 15)	14.3 ± 3.88		58.1	20.3 ± 3.22		83.4

*n* number of replicates, *No*° sample number, *SD* standard deviation

**Fig. 2:**
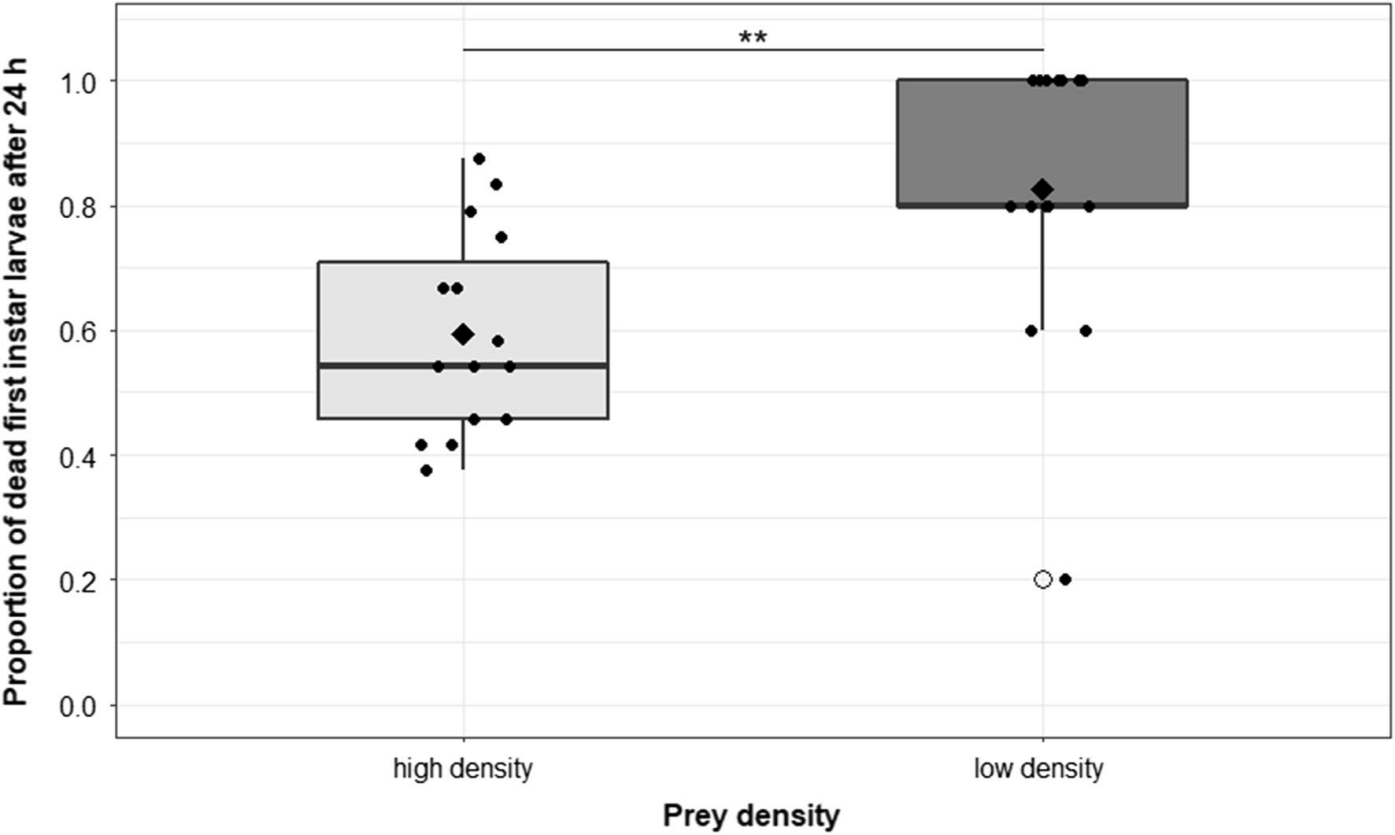
Proportion of dead *Ae. albopictus* first-instar larvae after exposing either 24 (high density) or five (low density) larvae per replicate (*n* = 15) to one adult copepod for 24 h. The rhombus indicates the mean of dead larvae in each group. Each point (filled circle) refers to one experimental replicate. Open circles represent outliers in the box–whisker plot. Statistically significant difference between groups, according to a GLMM analysis (***P < 0.01), is indicated by asterisks

The number of first-instar larvae killed after 24 h was found to depend on the prey density (five or 24 first-instar larvae, offered to the copepods at the beginning), and total consumption increased as more prey was supplied, but the proportion of killed larvae was significantly lower at the higher density relative to the lower density (GLMM model 1; *P* < 0.01; *n* = 15). In total, *M. viridis* killed more larvae when offered in higher densities, relative to when a lower density of prey was offered in the same volume of tap water (Fig. 2); however, the predatory efficiency was reduced.

### Predation efficiency analysis against different larval stages of *Ae. albopictus*

*Megacyclops viridis* did not display any predatory behaviour against *Ae. albopictus* second- or third-instar larvae when they were offered in the same way as for first-instar larvae (five larvae provided). No larvae of the further developed larval stages were eaten or attacked by the copepods, either after 24 h or after 48 h of predation, showing a clear preference for first-instar larvae.

### Simultaneous predation efficiency analysis against *Ae. albopictus* and *Cx. pipiens* s.l. larvae

**Fig. 3:**
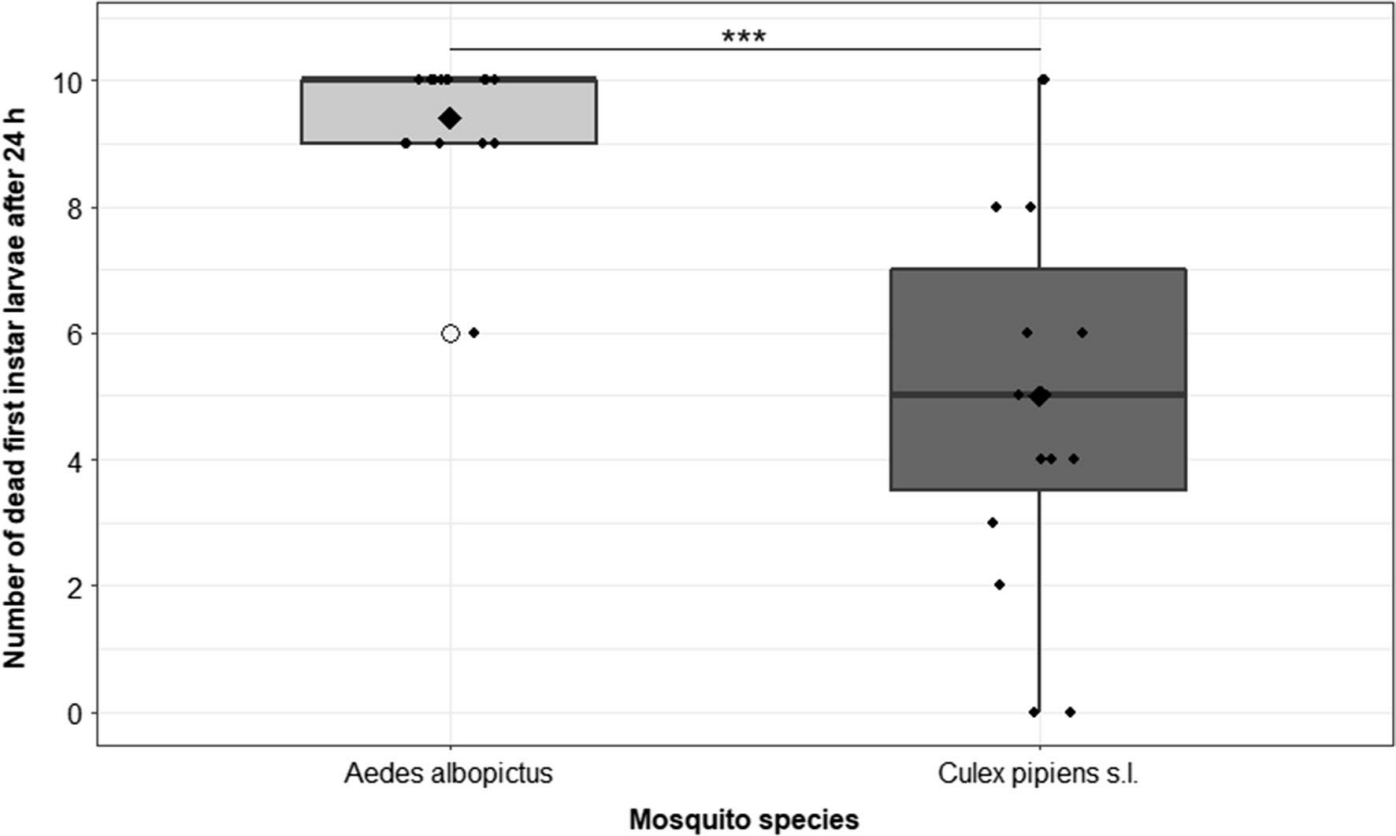
Number of dead *Ae. albopictus* and *Cx. pipiens* first-instar larvae after simultaneously exposing 10 larvae of each species per replicate (*n* = 15) to one adult copepod. Each point (filled circle) refers to one experimental replicate. The rhombus indicates the mean of dead larvae. Circles represent outliers in the box–whisker plot. Statistically significant difference between groups, according to a GLMM analysis (***P < 0.001), is indicated by asterisks

In the control experiments, 1.53 ± 2.33 of the initially inserted larvae died during the experimental period, whereas in the treatment groups with copepods, 14.4 ± 3.67 larvae died (not distinguished between mosquito species), indicating that larval mortality is based on the predatory behaviour of the copepods. Larval fragments were detected in the experimental containers as additional proof of feeding. The predation efficiency varied considerably, depending on the mosquito species (*Ae. albopictus* or *Cx. pipiens* s.l.) consumed (Additional file 1: Table S1). On average, *M. viridis* killed 9.4 ± 1.0 of the 10 *Ae. albopictus* larvae that were offered, but only 5.0 ± 3.1 of the *Cx. pipiens* s.l. larvae (Additional file 1: Table S1). All 10 *Ae. albopictus* larvae were consumed in nine out of 15 replicates. *Culex pipiens* s.l. larvae were also consumed, with strongly varying predation efficiency between the replicates (Additional file 1: Table S1). However, *M. viridis* showed a highly significant preference towards *Ae. albopictus* larvae (GLMM model 2*; P* < 0,001; *n* = 15; Fig. 3).

### Assessment of the compatibility of *Bti* and copepods in an integrated control strategy

The experiment revealed that *Bti* had no adverse effects on the copepods. Three applied concentrations of *Bti* (1, 10 and 50 ppm) killed all first-instar larvae of *Cx. pipiens* s.l. in every replicate (*n* = 5) of the positive control, whereas none of the copepods died or showed signs of reduced viability.

### Assessment of the predation rate of *Ae. albopictus* larvae in semi-field tests

The continuous detection of copepods and copepodids in the experimental barrels confirmed their survival and reproduction. Occasionally, the samples contained head capsules or torn larvae, proving the feeding behaviour of the copepods. Larval growth varied drastically, with most of the barrels containing all four larval developmental stages concurrently from the sixth day of the study onwards.

**Fig. 4:**
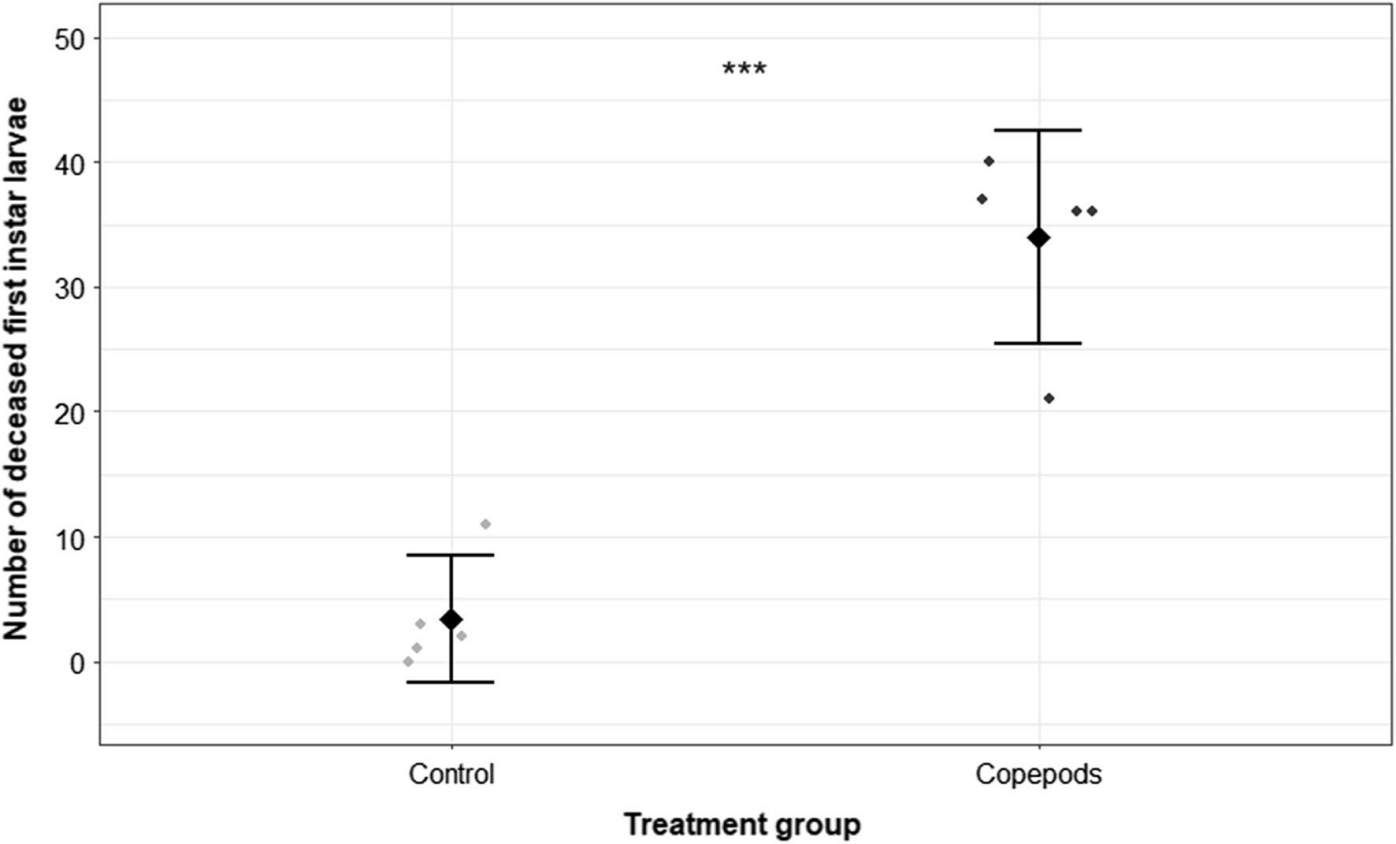
Number of dead *Ae. albopictus* larvae after exposing 50 larvae per replicate (*n* = 5) to 10 adult copepods under semi-field conditions. Results refer to the final evaluation day. Each point refers to one experimental replicate. The rhombus indicates the mean of dead larvae. Statistically significant difference between groups, according to a GLM analysis (***P < 0.001), is indicated by asterisks. Error bars represent the 95% confidence interval

In comparison with the control group, in which 3.4 ± 3.93 of the 50 initially inserted larvae died during the experimental period, the copepods significantly reduced the larval survival by killing an average of 34 ± 6.66 larvae (GLM model 3; *P* < 0,001; *n* = 5; Fig. 4). This corresponded to a predation efficiency after 7 days of 65.7%. Even though *M. viridis* reduced the number of surviving prey, up to 29 larvae still reached a developmental stage, in which they could not be killed, and would have potentially completed their growth (third-instar larvae). Most of the remaining third- and fourth-instar larvae were detected on the first evaluation day (fifth day of the study) and merely up to five larvae or fewer were counted at the third evaluation (seventh day of the study).

## Discussion

The capacity of some copepod species to behave as predators of various mosquito larvae favours their use as a component of an integrated control strategy in the fight against the Asian tiger mosquito *Ae. albopictus*. Copepods feed upon mosquito larvae by using mechanoreceptors, which detect the water motion caused by the prey when the larvae enter a radius of approximately 1 mM around the copepod. After successfully capturing the prey, the copepod uses its strong mandibles to chop the larvae into manageable portions and then ingests them into the oesophagus without further chewing [21]. Only the head capsule is left over, since this is the only part of the larvae which is already entirely sclerotised and cannot be eaten [1]. To ingest one single larva, the copepod requires only a few minutes, and the most efficient species are able to kill up to 30–40 *Aedes* larvae per day [20]. In addition, when more prey are present than they can ingest, the copepods have been observed killing the larvae using aggressive behavior, by attacking them and tearing out small pieces, producing residues of half-eaten and mutilated larval bodies in the containers, which was also observed in the present study [21].

Over the last few decades, different populations of the cyclopoid copepod, *M. viridis*, derived from various geographical regions, have been examined for their predation efficiency. Therefore, this copepod species is already known as an effective larval predator [21]. In fact, Russell et al. [30] have stated that *M. viridis* is more effective against *Ae. albopictus* larvae than the previously most widely surveyed and promising cyclopoid copepod, *Macrocyclops albidus* (*M. albidus*; Jurine, 1820), which has already proved to be effective under field conditions, successfully controlling *Aedes* populations in discarded tyres [27, 31, 34].

In our study, we were able to confirm the high predation efficiency of the native copepod *M. viridis* upon *Ae. albopictus* first-instar larvae, as observed in other studies [29, 30]. According to the results reported herein*, M. viridis* significantly reduced the larval survival relative to the controls, killing up to 4.8 ± 0.4 of the five offered larvae in a 24 h predation period. The copepods reduced the larval survival to an even greater extent after a predation period of 48 h. At this time, 4.2 ± 0.7 to 5.0 ± 0.0 larvae of the initial prey were killed, leading to high predation efficiency ranging from 84.0% to 100.0%. In accord with the findings by Rey et al. [31], in our study *M. viridis* did not prey upon any larval stage above first-instar larvae, as they exceed the maximum size of potential prey, emphasising the crucial importance of the size ratio between predators and prey for predation success.

When a higher density of prey (24 first-instar larvae instead of five) was offered to the copepods in the ad libitum feeding experiments, the number of killed larvae rose to 9–21 killed larvae per copepod in the first 24 h, and the overall predation efficiency within a 48 h predation period amounted to 87.0%, suggesting that a higher prey density increases the number of larvae eaten per copepod. Indeed, a significant difference between the experiments conducted here and those conducted by Dieng et al. [29] (where *M. viridis* killed 27 ± 2 larvae of *Ae. albopictus*) and Früh et al. [36] (where *M. viridis* killed 20–50 larvae of *Ae. japonicus*) is that, both times, an initial density of 50 larvae were exposed to the copepods, whereas the highest applied density under laboratory conditions in this study was only 24. Even though Russell et al. [30] calculated 24 as the number of initial prey at which the copepod predator is satiated according to a functional response analysis, strong evidence suggests that the predation efficiency of copepods increases with the larval density [31, 35]. This might explain the higher predation rates of other studies compared with those achieved here, and indicates that the prey density is a strong influencing factor regarding copepod efficiency.

The difference between the consumption rates in relation to prey density can be explained by the hunting behaviour of the copepods, which usually wait until they “bump” into the prey instead of actively hunting it [43]. As mentioned previously, copepods feed upon mosquito larvae by detecting their water motion, using mechanoreceptors. This allows copepods to detect larvae which are within ~1 mm. A higher prey density means a greater chance of copepods colliding with larvae, and hence the predation efficiency of copepods is most likely not limited by their ingestion rates, but by the struggle to locate them.

The same explanation accounts for the observation that predation efficiency is reduced when analysed in larger experimental volumes [44]. In fact, it has been observed that copepods kill 10–20 fewer larvae in large containers [22]. Most studies on *M. viridis* used small petri dishes with 20 ml [30] or even only 10 ml [36] of water, whereas 500 ml or more served as the experimental setting in this study. Both findings accord with the results of Micieli et al. [45], who also speculated that the cyclopoid species *Mesocyclops annulatus* (*M. annulatus*; Wierzejski 1892) was not able to reach high daily consumption rates with a prey density of under 50 larvae in 700 ml of water (simulating typical breeding sites) because of its inability to locate the prey in larger containers. As two conditions that have an adverse effect on copepod performance—a low prey density and a large experimental volume (5 or 24 larvae in 500 ml of water)—were both present in our experiments, and considering that *M. viridis* still induced high predation rates, the species can be presumed to be an effective larval predator.

Since *Cx. pipiens* s.l. is the most common mosquito locally that breeds near human settlements in similar breeding sites to *Ae. albopictus*, and is therefore very likely to be found alongside *Ae. albopictus* in the same containers, a comparison of the predation efficiency of copepods against both of these mosquito species is of interest. Copepods are known to display drastic differences in their prey preference, which was also confirmed in our experiments. When offering first-instar larvae of both mosquito species simultaneously to the copepods, the predation rate against *Ae. albopictus* amounted to 9.4 ± 1.0 killed *Ae. albopictus* larvae on average, in line with the promising efficiency of *M. viridis* against the Asian tiger mosquito in the previous experiments, whereas the predation rates against *Cx. pipiens* s.l. varied considerably, but generally always stayed below the feeding rates against *Ae. albopictus*. These findings confirm the results of previous studies, where *M. viridis* was shown to strongly prefer *Ae. albopictus* [35] or *Ae. aegypti* [46] over *Cx. pipiens* s.l. larvae.

These results are likely to be due to both predator and prey characteristics. *Aedes albopictus* feeds mostly at the bottom of containers (benthic feeders/browsers), where the copepods are commonly located, while *Cx. pipiens* s.l. mainly feeds at the water surface as filter/surface feeders [1, 47]. According to the feeding behaviour of the copepods, *Aedes* larvae are more likely than *Culex* to collide with the copepods and suffer attack, since their encounter opportunities are increased [45]. Additionally, *Ae. albopictus* are more active and display more persistent movements [48], creating stronger water motion near the copepods, and therefore increasing the chances of being detected by copepod mechanoreceptors, since copepods are only capable of catching moving prey [49].

Regarding several potential influencing factors, the efficiency of copepods against mosquito larvae had at this point only been evaluated in laboratory assays, and it was vital to verify these findings under more natural conditions, since copepod predatory behaviour can vary drastically under differing parameters, aside from the mosquito species. Therefore, a semi-field assay was performed in rain barrels, inoculated with 50 newly hatched *Ae. albopictus* larvae and 10 *M. viridis *copepods. The results reveal that larval mortality in barrels with copepods was significantly higher than that observed in control barrels, since 34 ± 6.66 of the larvae were presumably killed by the copepods over an evaluation period of 1 week (while only 3.4 ± 3.93 larvae died in the barrels without copepod predation).

At this point, the former explanation on varying copepod predation efficiency needs to be reconsidered. In other studies, a prey density of 50 larvae was occasionally exposed to copepods in 20 ml of water under laboratory conditions. In the present study, they were injected into approximately 52.5 L. Even though 10 instead of one copepod were introduced to the prey, it is very likely that the prey density within this volume was too small, so that the copepods had no chance to detect the larvae in the barrels. It could be ruled out that the lower predation rates resulted from the presence of further developed larval stages, since the copepods were injected to the barrels prior to the first-instar larvae.

The fact that the copepods still achieved a relatively high predation efficiency in our experiments under semi-field conditions underlines the premise of integrating *M. viridis* as a highly efficient predator in our control strategy to support the use of *Bti.* As expected, *Bti* does not harm copepods because of its highly specific mode of action. Additionally, the combination of both bioagents is thought to achieve a long-term effect against mosquito larvae. The application of *Bti* would kill all larvae at first, and as soon as the effect of *Bti* wanes, the copepods would feed upon all newly hatched larvae, since those (first-instar larvae) are their preferred prey. This approach would allow a lower frequency of *Bti* applications, hence a simplification of the door-to-door activities, and therefore the use of copepods alongside *Bti* applications are envisaged.

## Conclusions

The indigenous, field-derived cyclopoid species *M. viridis* proved to be highly efficient against *Ae. albopictus* larvae. Therefore, it is a promising candidate as a biological control that can be easily integrated as an additional eco-friendly element into a control strategy that is mainly based on the elimination and sanitation of breeding sites by community participation and the application of *Bti*. A prerequisite of the wide-scale use of *M. viridis* is the mass production of the copepods. However, there are some parameters of realistic environments that might limit its potential and should be considered in further examinations. This includes the ideal number of copepods applied to different volumes of water, their ability to survive in artificial containers during the mosquito breeding season and the possible adverse effect of the presence of alternative prey on predation efficiency.

## Supplementary Information

**Additional file 1: Table S1.** The predation efficiency against and number of consumed *Aedes albopictus *and *Culex pipiens* s.l. first-instar larvae, simultaneously exposed to *Megacyclops viridis* copepods.

## Data Availability

The datasets supporting the findings of this article are included within the article and its additional files.

## Abbreviations

*Ae*.: *Aedes*
*Cx*.: *Culex*
*M*.: *Megacyclops* or *Macrocyclops*
P4: Fourth thoracic appendage/paired swimming leg
P5: Fifth thoracic appendage/paired swimming leg
SE: Standard error
s.l.: Sensu lato
