# Supplementary material for: Efficacy of native cyclopoid copepods in biological vector control with regard to their predatory behavior against the Asian tiger mosquito, Aedes albopictus
Source: Parasit Vectors. 2022 Oct 1;15:351. doi: 10.1186/s13071-022-05460-y (PMC9526276; doi:10.1186/s13071-022-05460-y)
Supplement: Supplementary file 1 — Additional file 1: Table S1. The predation efficiency against and number of consumed Aedes albopictus and Culex pipiens s.l. first-instar larvae, simultaneously exposed to Megacyclops viridis copepods. [file 13071_2022_5460_MOESM1_ESM.docx]

| Table S1 Number of killed *Aedes albopictus* and *Culex pipiens* s.l. first instar larvae after exposing 10 larvae of each species per replicate (*n* = 15) simultaneously to one adult copepod. Statistically significant difference between groups according to a GLMM analysis (***, *p* < 0.001), is indicated by asterisks. *n*, number of replicates; No°, sample number; SD, standard deviation | | | | | | | | |
| --- | --- | --- | --- | --- | --- | --- | --- | --- |
|  | *Ae. albopictus* | | | | *Cx. pipiens* s.l. | | | |
| Run No° (*n*=5) | Dead larvae | Range | | Predation efficiency  [%] | Dead larvae | Range | | Predation efficiency  [%] |
| 1 | 8.6 ± 1.5 | 6 - 10 | | 85.1 | 2.8 ± 2.5 | 0 - 5 | | 23.4 |
| 2 | 9.6 ± 0.5 | 9 - 10 | | 95.3 | 5.8 ± 3.3 | 2 - 10 | | 51.2 |
| 3 | 10 ± 0.0 | 10 | | 100.0 | 6.4 ± 2.6 | 4 - 10 | | 62.5 |
| Overall  (*n*= 15) | 9.4 ± 1.0^***^ | | 93.5 | | 5.0 ± 3.1^***^ | | 45.7 | |
